# Context Matters: Findings from a Qualitative Study Exploring Service and Place Factors Influencing the Recruitment and Retention of Allied Health Professionals in Rural Australian Public Health Services

**DOI:** 10.3390/ijerph17165815

**Published:** 2020-08-11

**Authors:** Catherine Cosgrave

**Affiliations:** Department of Rural Health, Faculty of Medicine, Dentistry and Health Sciences, University of Melbourne, Docker St, Wangaratta, VIC 3677, Australia; ccosgrave@unimelb.edu.au; Tel.: +61-405-110-897

**Keywords:** rural health workforce, allied health, local context, recruitment, retention, turnover, Australia

## Abstract

Chronic health workforce shortages significantly contribute to unmet health care needs in rural and remote communities. Of particular and growing concern are shortages of allied health professionals (AHPs). This study explored the contextual factors impacting the recruitment and retention of AHPs in rural Australia. A qualitative approach using a constructivist-interpretivist methodology was taken. Semi-structured interviews (n = 74) with executive staff, allied health (AH) managers and newly recruited AHPs working in two rural public health services in Victoria, Australia were conducted. Data was coded and categorised inductively and analysed thematically. The findings suggest that to support a stable and sustainable AH workforce, rural public sector health services need to be more efficient, strategic and visionary. This means ensuring that policies and procedures are equitable and accessible, processes are effective, and action is taken to develop local programs, opportunities and supports that allow AH staff to thrive and grow in place at all grade levels and life stages. This study reinforces the need for a whole-of-community approach to effectively support individual AH workers and their family members in adjusting to a new place and developing a sense of belonging in place. The recommendations arising from this study are likely to have utility for other high-income countries, particularly in guiding AH recruitment and retention strategies in rural public sector health services. Recommendations relating to community/place will likely benefit broader rural health workforce initiatives.

## 1. Introduction

Many rural communities around the world struggle to attract, recruit and retain a full spectrum of health workers to service the often-complex health needs of diverse populations living in rural places. In Australia, these chronic rural health workforce shortages have been identified as significantly contributing to the substantial unaddressed health care needs found in rural and remote communities [1]. Of particular and growing concern are shortages of allied health professionals (AHPs), particularly given the greater reliance on collaborative, team-based care in rural places and the lead role that AHPs play in providing rehabilitation and chronic disease management services [2,3]. Shortages of AHPs are also likely to be a contributing factor in the lower hospitalisation rates for rehabilitation care among Australians living in rural areas, with 6.9 hospitalisations per 1000 population for outer regional areas and 6.2 for remote areas compared to 19 in major cities [1]. Allied health (AH) rural workforce shortages also persist despite substantial investment by the Australian Government over the last 20 years in funding university places to increase the number of trained AHPs [4,5].

The rural AH workforce issue is primarily one of maldistribution, with an oversupply of AHPs in metropolitan areas and an undersupply in rural areas, especially of experienced AHPs, with AH workforce shortages intensifying with remoteness [1,6,7]. In 2017, 81% of physiotherapists, 75.5% of podiatrists, 77.1% of occupational therapists, 77.4% of pharmacists and 79.1% of medical radiation practitioners worked in major cities, while only 72% of the Australian population of approximately 25 million lives in major cities [1,5]. In rural and remote Australia, there is also greater reliance on the public health sector given there is more limited access to private health services [8]. Related to this, studies indicate that approximately half to two-thirds of rural AHPs work in public sector services [9,10]. In an Australian study in western Victoria that measured rural AH workforce turnover and retention, career grade was found to influence retention, with AHPs at Grade 2 or higher having a significantly reduced risk of leaving their rural position compared to those who commenced at Grade 1 [6]. The grade level classification system for AHPs working in the public sector is outlined in the relevant Australian state or territory’s Enterprise Bargaining Agreement (EBA) for AHPs. An EBA is an agreement, made at an enterprise level between employers and employees and their union, about terms and conditions of employment. Grading of AHPs starts at Grade 1 for new graduates, rising to Grade 3–4 for managers. The classification level relates to the degree of responsibility, skills and experience for the position, not an employee’s performance in the position. The western Victoria study also found that being under 35 years of age at commencement of employment had an important and statistically significant association with turnover risk [6]. The authors attributed this higher risk of turnover among younger, entry-level AHPs to the limited opportunities for grade level advancement, given the small size of AH workforces found in rural and remote health services [6]. Thus, to improve the stability of the AH rural workforce, recruitment and retention strategies need to address the particular factors underlying the different turnover risks between early career and more experienced AHPs.

While the AH rural workforce problem is well recognised [11], the development of effective recruitment and retention strategies to support the achievement of a stable and sustainable AH workforce in rural places remains elusive. In part, this can be attributed to the complexity of the issue, with the reasons why health professionals’ come, stay or leave a rural position being multifaceted, involving personal, organisational, social and spatial aspects that change over the life course [12]. In addition, for recruitment and retention strategies to be effective in rural settings, as well as being evidence based, they must be context specific and founded on a sound understanding of the unique factors at play in each service and place [13]. In response to this complexity and the need for a person-centred, evidence-based and context-informed approach, the author developed a rural health workforce conceptual framework—the Whole-of-Person Retention Improvement Framework (WoP-RIF)—to support Australia’s rural health service executives and line managers, rural communities, and governing bodies to develop effective strategic actions to improve rural health workforce retention [14]. While the WoP-RIP’s focus is on retention, it does not ignore recruitment or attraction. Rather, retention is conceptualised as starting with recruitment and the importance of ‘person-environment-fit’ selection is emphasised with attraction as a key component of recruitment [14].

### Guiding Theoretical Framework

**Figure 1:**
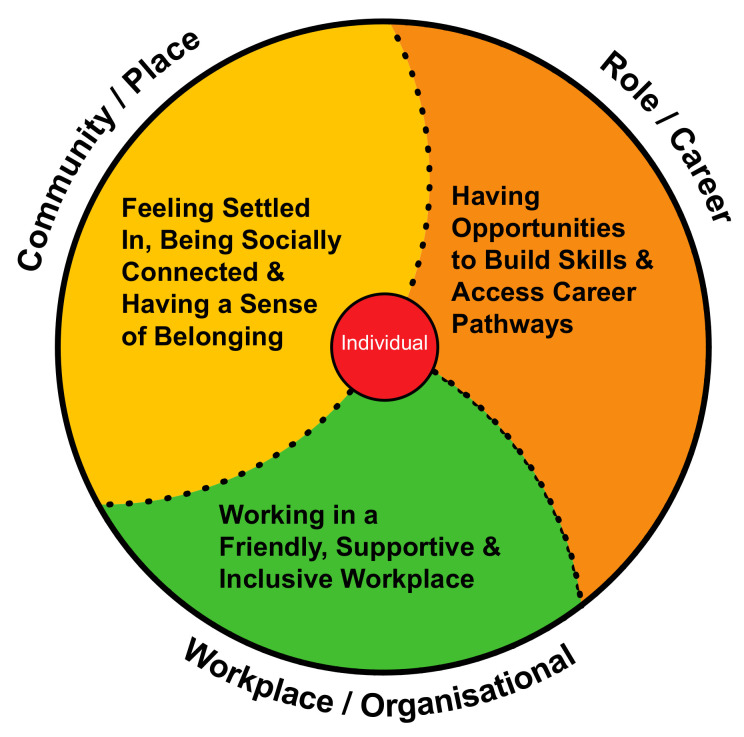
The Whole-of-Person Retention Improvement Framework.

In this paper, the WoP-RIF is drawn on to help guide an exploration of the service and place factors influencing recruitment and retention of AH staff working in rural public sector services. For this study, the WoP-RIF was used to inform the development of the participant interview schedule, thematic analysis of the interview data, and the development of the retention improvement recommendations for each service. The WoP-RIF resulted from the author’s grounded theory study investigating the full range of ‘life’ factors influencing the turnover intention of AHPs and nurses working in rural public health services in New South Wales, Australia. It was developed from a substantive theory explaining turnover intention, which was then cross-referenced with the extensive body of rural health retention literature [14]. The WoP-RIF has three domains—workplace/organisational, role/career and community/place—and the necessary preconditions for improving retention through strengthening job and personal satisfaction are set out under each domain. These preconditions are: working in a friendly supportive, inclusive workplace (workplace/organisational); having opportunities to build skills and access career pathways (role/career); and feeling settled in, being socially connected, and having a sense of belonging (community/place) (see Figure 1).

The major known influences regarding the job and personal satisfaction of rural-based health staff under each domain were explained in a paper detailing the WoP-RIF [14] (see Table 1).

## 2. Materials and Methods

### 2.1. Aims

The aim of this study was to explore the contextual factors perceived and experienced as impacting the recruitment and retention of AHPs amongst executive staff, AH managers and newly recruited AHPs working in rural public health services. This research was the first stage in a larger project seeking to produce new knowledge about how rural-based public health services can better attract and improve the retention of AHPs through the implementation of a set of evidence-informed and contextually-specific recommendations. This research was guided by the following research questions:

- How do AH staff working in rural public health services perceive their current work and personal experience as influencing job retention?
- What are the service and place-specific challenges and opportunities facing rural public health services in achieving a sustainable AH workforce?

This study takes a broad definition of AH and AHPs, drawing on the Allied Health Professions Australia’s description on its website: AHPs are qualified health practitioners with specialist skills in preventing, diagnosing and treating a range of conditions and illnesses. This study also draws on the Victorian Government, Department of Health and Human Services’ (DHHS) categorisation of AH, which includes health professionals from the therapies (including AH assistants working under the supervision AH professionals) and the sciences. In regard to the geographic setting, given the strength of existing evidence that retention challenges increase with remoteness, all areas outside major cities were of interest [1]. Herein, the use of the term ‘rural’ includes regional and rural places unless otherwise specified. Ethical approval for this study was applied for and granted by The University of Melbourne’s Department of Rural Health Human Ethics Advisory Group (1749205).

### 2.2. Design

This study was undertaken in Victoria, the smallest state on Australia’s mainland, the second most populous and the least geographically remote state or territory in the country. This study was conducted with AH staff from two rural public health services. Victorian public health services are state funded through the DHHS. Under the DHHS’s health service classification system, the two health services selected were a regional service and a medium-sized rural service, hereafter referred to as the regional health service (ReHS) and the rural health service (RuHS). These two service types were selected to support a full exploration of AH staffing challenges in rural services, which are known to differ not only due to remoteness but also in relation to service size [15].

The two health services selected were identified by drawing on the University of Melbourne, Department of Rural Health (UoM-DRH) research team’s knowledge of rural Victorian public health services, an assessment of the services’ AH workforce challenges, and the perceived level of likely interest by the services’ executive and senior AH management in partnering with UoM-DRH to undertake this research project. The UoM-DRH is one of 16 DRHs funded by the Australian Government to undertake multidisciplinary rural health education and research with local communities in a specified geographical footprint to address unmet health care needs. The UoM-DRH footprint is within rural Victoria.

A legally binding project partnership agreement was drawn up between UoM-DRH and each of the two participating health services. Each agreement outlined partners’ cash and in-kind contributions, project inputs and governance arrangements. The main financial input was the employment of a project worker in each service. For this stage of the study, the project worker assisted the author in recruiting staff participants and developing retention improvement recommendations. The governance arrangements included the operation of two groups for the duration of the full study: the Project Working Group (PWG) and the Project Reference Group (PRG). The PWG members included the author, an assigned senior AH manager and the project worker at each site. The PRG members included PWG members, the UoM-DRH Director, senior AH staff and executives of the particular service. In the regional service, a community representative from the local council was also a member of the PRG.

To identify the contextual factors impacting the recruitment and retention of AHPs, a qualitative approach using a constructivist-interpretivist methodology was taken in this study [16].

### 2.3. Participants

This study used a purposive sampling method to recruit participants for semi-structured interviews who were either 1) AHPs in ‘early career’, 2) ‘experienced’ AHPs who had relocated for work within the last 12 months, or 3) key informant staff members on the AH workforce. From this point in this paper, 1) and 2) will be described as ‘target AH staff‘ and 3) as ‘key informants’. In this study, ‘early career’ was defined as having worked less than three years in an AH health role since graduating and ‘experienced’ as working for three years or more. In the ReHS, the target AH staff participants included both the therapies and sciences. These staff worked at various sites in a broad range of teams and settings including inpatient and community. In the RuHS, the target AH staff participants were mostly from the therapies, working from one site and treating both in- and out-patients and community. The AH workforce is generally considered to exclude medical, nursing and dentistry. However, the RuHS requested that dentistry professionals be included for this study given that a dentistry service was co-located with AH and considered to be part of community services.

### 2.4. Recruitment

To recruit participants, the two project workers made presentations at AH team meetings and management meetings and/or discussed the project with individual potential participants. Staff members interested in participating gave the project worker permission to share their work email with the author who then made contact, inviting the individual to participate in a face-to-face interview and attaching a copy of the plain language statements (PLS) and consent form (CF). The author liaised with each participant to identify a suitable time to schedule a face-to-face interview. All interviews were conducted at the health services in a pre-booked room selected, whenever possible, at a distance from ‘usual’ AH work areas to reduce the risk of participant identifiability.

### 2.5. Interview Data Collection

The author brought hard copies of the PLS and CF and, before each interview, any questions were answered, and consent was given by the participant signing the CF. The interviews were scheduled for one hour and most interviews were between 45 and 60 minutes in length. A flexible interview guide was used to focus the conversation. For the target AH staff group, this included questions on reasons for taking the position, onboarding experience, quality of relationships with line manager and team, extent of job satisfaction with role, access to professional development and career development opportunities, perceived social connection in the workplace and in-community, and personal satisfaction with the local community and place. For those target AH staff participants who had relocated to take up their position, additional questions were asked to explore their experience of relocating and the perceived level of organisational and community support received. The key informant participants were asked questions regarding their experience of attracting, recruiting and retaining AH staff and their perception of the organisational, team, community and place challenges and opportunities for achieving a sustainable AH team/workforce. The interviews were audio-recorded and handwritten notes were taken during the interview to assist the author with participant recall and identifying important aspects of the discussion during analysis.

### 2.6. Interview Data Analysis

The audio-recordings were transcribed verbatim into separate Word documents and then checked by the author for accuracy against the original recording. The author assigned a unique identifier to each transcript denoting the service type: regional (ReHS) or rural (RuHS); the participant type: target AH staff (TAHS) or key informants (KI); and the interview number for that service and group type (e.g., ReHS-KI-6). The author then conducted a thematic analysis of the data using NVivo v12 software (QSR International) [17]. The WoP-RIF’s three domains—workplace/organisational, role/career and community/place—provided structure for the first level of analysis—coding and categorisation. Identification of emergent themes drew on the key influences on staff job and personal satisfaction under each WoP-RIF domain (see Table 1). These identified themes underpinned the development of a set of recommendations for each service to support a sustainable allied health workforce. Fourteen recommendations were made for the ReHS and 13 for the RuHS; 10 of these recommendations were common to both services. This paper focuses on the data underpinning these 10 shared recommendations because these are most likely to resonate with, and have utility for, other rural health services.

### 2.7. Rigour

Within NVivo data coding, listing explaining preliminary codes, categories and themes was developed to support consistency in the coding. The thematic coding was checked for consistency by another experienced qualitative researcher and minor adjustments were made. Given that no conflicts or uncertainties arose, a third reviewer, who would have been consulted if there were discrepancies, was not sought. To safeguard participant confidentiality, the transcripts were not shared with any other PWG or PRG members. The author only ever presented the findings thematically with deidentified supporting quotes. To check the rigour of the author’s analysis, a presentation of the findings was made to senior executive staff and/or PWG members in each service and these members expressed that the themes identified were relatable. A set of draft recommendations were then developed, initially by the author, and then worked on by all members of the PWG. These recommendations were then presented to the respective PRG for approval. Formal approval to implement the recommendations was given by the PRG in the regional service in late May 2018 and the rural service in June 2018.

## 3. Results

### 3.1. Overview of Participants

Interviews were conducted by the author from November 2017 to May 2018. A total of n = 74 participants were interviewed—n = 53 from the regional health service (n = 37 target AH staff and n = 16 KI); and n = 21 from the rural health service (n = 14 TAHS and n = 7 KI). See Table 2 for an overview of participants.

The key contextual factors perceived and experienced as impacting the recruitment and retention of AHPs identified in thematic analysis are presented under each of the WoP-RIF domains: workplace/organisational, role/career and community/place. A summary is provided in Table 3. In the last domain, ‘place’ is discussed before ‘community’ as finding suitable accommodation was found to be the initial primary need that participants relocating for work needed to address before social connection was prioritised.

### 3.2. Workplace

#### 3.2.1. Degree of Challenge: Attracting, Recruiting, and Retaining Allied Health Professionals (AHPs) Varies Depending on Profession, Experience Level and Life Stage

The managers interviewed in both services were all familiar with the challenges of recruiting and retaining AH staff. Most executives and AH managers demonstrated a nuanced understanding of the different factors impacting the recruitment and retention of AHPs. Managers discussed the differences between AH professions in terms of the degree of challenge for recruiting. Dietetics and exercise physiology positions were commonly discussed as being fairly easy to recruit to, given an oversupply of graduates, while physiotherapy and occupational therapy, the latter especially since the commencement of the Australian Government’s National Disability Insurance Scheme, were described as being challenging to recruit to: ‘They’re in oversupply, there’s no physios and no podiatrists, but there’s too many dietetics.’ [RuHS–KI-4]

This observation was supported by new staff participants, with those in over-supplied professions often attributing their motivation for taking the position as primarily being just wanting to get some work experience: ‘I was looking in Melbourne as well but there weren’t a lot of openings’ [ReHS-TAHS-25]. In the regional service, this group of staff were often on fractional short-term contracts and many were commuting, sometimes long distances, or staying over on workdays. The cost of this travel and/or accommodation was borne by the individual staff member:

So, I commute every day [it’s] one hour and 20 [drive] to here. Yeah, I do 240kms a day when I work here. I only work here three days a week now.

*ReHS-TAHS-6*

Managers commonly observed differences in recruitment and retention challenges between early career AHPs compared to more experienced AHPs. Recruiting to entry-level positions was described as being achievable, ‘It is far easier to attract a Grade 1’ (ReHS-KI-3), but retaining them beyond 12 months was considered challenging.

I know I can keep my staff for 12 months. I can do that. I’ve got systems in place that they’re that busy and they’re that well-nourished and they’re that supported that 12 months is easy. 12–24 months, it’s gets a bit more tenuous.

*ReHS-KI-4*

This high turnover among early career AHPs was explained by one participant as being the result of skills development plateauing after the first year or so of rural practice:

The benefit of them being here in terms of skills development are huge in the first year, probably pretty solid in the second year, I don’t know what they would gain in clinical advantage being here after two years, so …that third year would really be more for the community’s benefit than for them … they’d need social reasons to stick around.

*RuHS-KI-6*

Most managers considered the high turnover among early career AHP staff as unavoidable and it was commonly attributed to social determinants, especially relationships, as well as the common desire among young adults to seek new experiences:

The single thing that will bring it all down, is the social side.

*ReHS-KI-4*

It’s ongoing. There seems to always be a constant flow of vacancies but not for the wrong reasons, it’s … that younger group heading off overseas … heading off to the next opportunity.

*ReHS-KI-10*

Managers generally described attracting ‘experienced’ AHPs to relocate for a rural position as being difficult:

I think one of our big recruitment areas is the Grade 2s in our community teams—getting someone who’s mid-career to come here for that next step in their career.

*ReHS-KI-8*

One manager attributed these difficulties to the barriers posed by life stage and social factors by older, more senior AHPs:

When you are mid-career, when you’ve hit a Grade 2 level, you usually have been working for a little while, you’ve sort of set your group somewhere. You’re not necessarily going to up and shift … you might shift for a partner, but you won’t necessarily shift for a job.

*ReHS-KI-3*

Both health services were situated in towns that were considered by many managers as being difficult to encourage longer-term stays by early career AH staff, particularly those who were partnered:

The partners aren’t interested in coming up to X [town’s name], it’s not geographically a big enough drawcard that they could envisage themselves living up here.

*ReHS-KI-4*

In the regional service, high AHP turnover was also connected to staff being on short-term contracts and/or part-time/fractional appointments and leaving to take up more secure employment in another health service, either a permanent and/or full-time position. This particularly involved AHP staff who were on maternity leave contracts:

I’m Grade 2, so if you want to retain that next level of workers—kind of the middle seniority—you have to give them permanent hours … because it’s just too difficult to be going from contract to contract.

*ReHS-KI-5*

In the rural service, to make health positions more attractive, most AH positions were offered as permanent, full-time roles, and if relocation was required, eight weeks of minimum transitional accommodation and reimbursement of relocation costs up to $1,000 was provided. In addition, recognising that a six-month probationary period made it difficult for new staff to secure private accommodation in the town, the service had recently shortened the probation period from six months to eight weeks. These employment incentives were appreciated by new staff and described by some as influencing their decision to take the position:

On the contract, on the letter, they already say they are going to supply eight weeks of free accommodations. …[The offer of accommodation was] very important, probably the most important fact, because like for me, because I’m living in Melbourne… it is actually impossible for me to get any accommodation because I don’t want to get accommodation I haven’t seen before I rent it. So, it’s very important for that … accommodation.

*RuHS-TAHS-13*

The hospital provided it [accommodation]. They’ve got quite a few houses around that they lease off landlords or whatever and then they charge people to come in. But they provided accommodation for X weeks free, no bills, no nothing. I thought that was magnificent, that was a really attractive thing coming down here…free accommodation and really good accommodation too.

*RuHS-TAHS-7*

Constant AHP staff turnover was discussed by most managers from both health services as having both direct and indirect costs, the latter particularly relating to the burden placed on other AH team members in terms of the time taken to orientate new staff and the extra workload carried while positions were vacant and new staff members were getting up to speed:

[We are] turning over positions every six months. By the time they’re orientated and can start being useful, they’re actually leaving. So, it’s not a good result for the community and it’s not a good result for us financially either, having to run and educate and it’s demoralising for other staff to have to orientate and onboard, and at some stage you get fatigued with that.

*RuHS-KI-4*

#### 3.2.2. Allied Health (AH) Managers Usually Recruited from Existing Workforce and Poorly Prepared for Leadership

In both services, the skill level of AH managers was described as variable, with some managers considered exemplars and others having a poor level of understanding and skills:

I’ve been told by a few others that I have a softer style that helps to try and nurture and bring people along—not as direct as some might be. So, having that open-door policy and so forth to make sure that they’re comfortable, they can come and talk at any time. So, it’s about being open, being honest with them, answering their questions, helping to guide and support them.

*ReHS-KI-10*

My manager creates the environment and I feel like… she’s the very key reason the staff that I work with are here and a very key reason for why I love to work here.

*ReHS-TAHS-6*

Some managers discussed feeling that they did not have the requisite skills or experience to be good managers:

Everything [staff member’s name] says she wants, I never got at her stage, so I don’t know what it looks like. I’ve never had a mentor. I’ve never been supervised.

*RuHS-TAHS-3*

Given challenges attracting Grade 2 and higher-level staff, AH managers were often recruited from the existing pool of clinical staff who had stayed on and eventually been promoted to managers. The level of management skill by these new managers was commonly considered to be poor:

From workforce perspective that was a concern … I had people that were being remunerated as seniors that weren’t necessarily acting or taking responsibility of seniors, not all of them.

*ReHS-KI-2*

This low skill level was explained by one manager as relating to the focus of AH university training:

You don’t go to uni to learn to be a manager, you go to uni to learn to be a clinician, the rest is on the job.

*ReHS-KI-10*

In both services, some staff and KI mentioned that AH managers needed more training and support, especially new managers:

I think for early leaders it’s a real challenge… that’s hard and that’s where I think [name of service] is off track it needs to support those new emerging leaders as they come into those roles.

*ReHS-KI-10*

### 3.3. Organisational

#### Overly Complex Human Resources Systems Negatively Impact Successful AHP Recruitment and are Burdensome for AH Managers

In both services, human resources recruitment processes were mentioned by many AH managers and some new staff as compliance focused and overly complex, resulting in onboarding delays being commonplace:

Our HR department seems under resourced. The responsiveness to getting our staff on board, they’re off accepting another opportunity before [HR have] managed to complete an onboarding or even to get to onboarding. It’s a challenge, 2–3 weeks to get back to someone to say ‘Yeah, you’ve been through all those processes and you are now successful’. That’s a long time.

*ReHS-KI-10*

A few managers attributed slow onboarding to their having lost their preferred candidate:

We have major issues with HR … It’s killing us … So, the guy that was due to start today … he still didn’t have a contract 10 days beforehand and I recruited him six weeks ago. And [so] you lose them. I just wonder if he had had a contract and signed it whether he would have felt committed?

*ReHS-KI-15*

Many new staff, particularly in the regional service, also mentioned experiencing longer than expected delays during the onboarding processes resulting in their becoming concerned about the soundness of the employment offer:

I understand there was a bit of a HR block here. So the HR process took a long time to come through … Maybe I interviewed in early Feb then, because I remember starting on the [late date in] March … as that was as soon as HR could onboard me … So I remember like it made me doubt myself … and I thought how could I have not gotten this job?

*ReHS-TAHS-10*

Given the high turnover of allied health workforce, most AH managers fairly continuously discussed having to recruit staff, particularly in the regional service, and the delays in human resources processes increasing their workloads:

My recruitment efforts have been enormous but for every recruitment, the amount of time I have spent riding HR to get things through has meant there’s 10 other things I’m not getting done.

*ReHS-KI-15*

In both services, managers often mentioned feeling stressed or were described by their staff or other managers as being stressed. This was discussed as negatively impacting team morale and individual staff members, particularly new graduates:

I think reducing the stress and burnout on the senior clinicians. There’s some teams at the moment where I think the stress levels of the senior clinicians is not a great environment for the new grads to be in at all.

*ReHS-KI-8*

### 3.4. Role

#### 3.4.1. Most Entry-Level AH Staff Experience a Challenging Adjustment

Almost all entry-level AH staff discussed experiencing a challenging initial adjustment to work, feeling both overwhelmed by the size and demands of the job and lacking confidence in their clinical skills and decision making:

So, the X [name of the clinical team] area is incredibly fast paced and busy and there isn’t a lot of time to think, prepare, discuss. It’s bang, bang, bang, and bang. … Absolutely [it’s a] ‘do’ job, [there’s] very little time. The culture is reflected in that, that everybody’s very efficient and busy and quick and there’s not a lot of sort of chatting.

*ReHS-TAHS-7*

Some entry-level AH staff attributed their initially low level of job satisfaction from the pressure they had put on themselves to quickly get up to speed so they could share the workload:

I got to the point after three months where I was booking in, probably overbooking a little bit, because I’m like, ‘Oh, the wait list is huge, I’m going to try and get through, try and get through it’. And then things would pop up on the IPU (inpatient unit) that were urgent, and I was getting quite stressed because I couldn’t fit everything into the day.

*RuHS-TAHS-4*

The benefit of having a supportive manager and team to help navigate the adjustment to the workplace and the workload and in building clinical confidence was mentioned by several entry-level AH staff:

Anything I need, anything I have to run by them, they make the time for me and X [name of manager] really gives me a lot of confidence in my abilities. She’s like, ‘Why are you worrying about this? It’s exactly what I would have done.’ ‘Of course, you’re on the right track.’ ‘If you forgot to ask a question [to a patient], you can go back and see them, tomorrow, can’t you?’ or ‘It’s just no fuss.’ I’m stressing about these things that I was made to stress about on placement which I don’t ever stress about here, it’s completely different.

*RuHS-TAHS-9*

On the other hand, the perceived absence of a supportive manager was sharply felt and described as having negative impacts on job satisfaction:

[Early career is] not really easy. I personally don’t advise new grads to work in rural anymore. I think they need support and no matter how much promise they get, I got a lot of promises but I didn’t get a lot of support.

*RuHS-TAHS-1*

While the extent of work challenges for entry-level AH staff were similar in both services, the different sizes of the two services posed distinct challenges, benefits and opportunities. The community health team in the rural service mostly comprised small AH discipline-specific teams (of 2–3 staff) and some solo practitioners. The small AH team sizes meant that entry-level staff in a team were highly dependent on their discipline-specific line manager’s skill level and interest in supervising and mentoring. It was well understood by the executive and AH managers in the service that new graduates working as solo practitioners had a heightened turnover risk and as a result, the service had implemented a number of strategies to try to reduce the risk. These strategies included increasing one of the AH teams full time-equivalent staffing to two, organising, during the recruitment phase, external supervision with a discipline-specific AHP from a nearby regional service; the community health service manager taking line management responsibility for the solo practitioners and organising weekly or twice-weekly catch-up meetings; and placing solo practitioners in shared offices. The service also encouraged entry-level AH staff to participate in a 10-month program for early career AHPs that was being run annually by another health care service in the region and within a one-hour driving distance.

The much larger AH team sizes in the regional health service, at least in theory, afforded entry-level staff access to both a discipline-specific line manager and team members. Some new entry-level AHPs spoke highly of the level of support that other team members gave them in the adjustment period:

[It’s] the best team. … so approachable [and] non-judgemental, because I come up with some stupid questions sometimes. But [they’re] just very, very supportive. Willing to go the extra mile, to kind of make you feel comfy or address any issues or whatever. If you’re like, ‘Oh, could I talk to you about this?’ They’ll go, ‘No, no sit down, what’s happening?’ … My manager creates the environment and I feel like … she sets the culture.

*ReHS-TAHS-6*

On the other hand, some entry-level AH staff experienced their team as unsupportive and described this as adding to their adjustment challenges:

Well, there’s a bit, maybe bullying might be the wrong word, like it’s not as strong. But I just feel like the people who have been here a lot longer, when there’s new people who come, the expectations they have of them are very high … When they’re … doing that same shift, they … expect that newer persons to have done all those things … [that] they themselves they don’t usually do.

*ReHS-TAHS-24*

The AH management in the regional service recognised that entry-level staff have particular adjustment needs and, in response, the incumbent in the Allied Health Educator position [this position did not exist in the rural service] had established a support group for early career AH staff which, at the time of interviewing, had been operating for a couple of years. This support group involved monthly face-to-face sessions on specified topics that had been selected based on the expressed needs and interests of the attendees. However, awareness of this group’s existence was fairly low among many AH managers and AH staff, especially in the sciences. Many entry-level staff discussed that even if they had been were aware of the group, it would be difficult for them to attend given their team’s heavy workloads and staffing shortages, and so they did not think their managers would support their attendance.

#### 3.4.2. Professional Development Opportunities Are a High Priority for AHPs and the Level and Type of Support Offered Is not Always Well Understood by AH Staff or Consistently Implemented by AH Managers

Access to, and organisational support for undertaking professional development (PD) was important to all new AH staff, and especially among those in early career. In both services, what PD was available and what external PD would/could be supported by the organisation was often unclear and applied differently by AH line managers. In the rural service, new AH staff were generally satisfied with the amount of external PD they had access to, and they described their PD requests as nearly always being accepted, and the service covering their salaries as well as paying for the training course and any accommodation and travel expenses.

I had a little bit of interest in learning [a particular discipline-specific approach] … and it’s something you [the service] might offer in the future, but probably not anytime soon. But I wanted to do it for my own sort of learning and interest. And work was supportive of me taking the time off for leave and paid for the course as well, which I really didn’t expect. Which was really nice, and the course was run over a Friday and then a Saturday morning and they offered either time in lieu or to be paid for the Saturday morning as well, which I didn’t expect. Because I was happy just to, so yeah, they were very, very, very supportive.

*RuHS-TAHS-4*

One of the things that I thought was really important for me when I wanted to come and work here was about the opportunity for professional development and ongoing learning … That’s really important to me. I don’t know, we’re all learning people, that’s why we go into this lifestyle and X [health service’s name] have been really good.

*RuHS-TAHS-8*

Senior management viewed the service’s current PD system as a free for all and urgently in need of a more strategic and systematic approach:

I came from X [another rural service’s name] and you got $250 a year [for external CPD], that was it. And here we’re paying thousands and they’re going off to all sorts of things and even for locums. [In my head] I’m going, ‘Oh my god’, but I haven’t [changed anything yet]. I’d like a framework so that I can be transparent in decision making and equitable [regarding funding for external courses]. So, if you can produce one of those that would be fantastic.

*RuHS-KI-2*

In the regional service, AH staff had a range of experiences regarding PD support and these seemed to vary depending on their expectations, how PD support was presented during recruitment, and then later supported by their line manager:

There’s no [financial support for PD], you just get the leave … And it wasn’t made clear [during recruitment]. I only found out from someone here who said, ‘[It’s] in the EBA that there’s no money’.

*ReHS-TAHS-36*

Yeah, good training opportunities, quick training opportunities, you’re able to get training quickly here as in compared to bigger metropolitan cities [where] it takes a while.

*ReHS-TAHS-35*

The regional service’s PD support was in line with the EBA for Victorian public sector AHPs, where full-time staff are entitled to five full days paid leave (pro rata for part-time staff) excluding any mandatory training, and all staff, both full and part-time, are entitled to two days‘ paid study/conference/seminar leave. One manager felt that AH staff access to PD was ‘pretty good’ and ‘probably more so than if they were [in] metro services’:

We have a budget. My budget’s $500 for the year for the whole team … What I do is, I say to them, ‘I’m very supportive of professional development, you tell me what you want to go to, and if you will be prepared to pay for it [up front, then] we’ll apply for a scholarship through RWAV [Rural Workforce Agency Victoria], and that’s usually not knocked back and the organisation pays for the days.

*ReHS-KI-3*

However, other managers felt that the pre-EBA-RWAV system, when the service had its own budget for PD support and was able to directly fund staff to attend courses, had been important for attracting candidates:

Many years ago, the hospital funded lots of stuff and it was fantastic, and it was a great drawcard and it was really good. It was like, ‘Yeah, we’ll fund you for a course a year maybe’. They’ve pulled all that back.

*ReHS-KI-4*

Having some budget for PD support and flexibility in managing was considered by one manager as being especially important for attracting Grade 2 and above AH staff:

I think, recruitment wise, focusing on how we market that [PD support] and what incentives we offer to people at that stage of their life to be taking that next career step here.

*ReHS-KI-8*

### 3.5. Career

#### Limited Career Development/Advancement Opportunities for AHPs Working in Rural Services

It was generally thought, by both staff and managers in both services, that there were few opportunities for upwards career development. In the rural service, this was related, in part, to the EBA establishing a maximum grade level of Grade 2 for medium and small rural services. AH staff participants also mentioned low turnover of senior roles and little growth in the service:

There’s two opportunities [at the moment] but then if they … get two people and they are [then] here for 30 years, you’re going to be stuck as a Grade 1 for 30 years.

*ReHS-TAHS-24*

There’s not much movement within organisations as well, particularly when it’s a smaller organisation. It makes it harder because there’s not as much growth usually.

*ReHS-TAHS-1*

If there were the opportunities to step up, yeah absolutely [I’d stay]. If there’s not, then I’ll leave.

*RuHS-TAHS-5*

Managers also recognised the importance of there being career opportunities for retaining staff:

When it comes to retaining them, well I think we have touched on it, in regard to the opportunities that they have to grow and develop.

*ReHS-KI-10*

### 3.6. Place

#### Securing Suitable Housing is a Priority Issue for all AHPs Relocating for Work

Almost all AHPs in both services who relocated to take up a position in the health service described having similar establishment needs. Primarily, these related to finding suitable housing, making friends and finding activities of interest, with housing being the primary initial concern.

Both the towns experienced chronic rental housing shortages:

I didn’t realise how hard it was going to be for them to get housing and, in hindsight, I probably should have.

*MN ReHS-KI-4*

However, housing shortages impacted differently in the two services. As discussed above, the rural service routinely provided a minimum of eight weeks paid transitional accommodation and newcomers generally described management as being highly supportive in regard to assistance with housing. Given the relatively small AH workforce, AH managers and executive staff were usually aware of where new staff were up to with finding suitable housing and would step in to support staff if obstacles were encountered. New staff who were having difficulties finding private suitable housing discussed being allowed to stay longer in the services’ transitional housing while paying a significantly under-market rent, the lease on a transitional house being handed over to staff member(s), a departing manager organising the transfer of their lease to two new staff members, and being assisted, from the outset, by management to find housing suitable for pets.

Everyone was very supportive, and you know [saying], ‘I’ve got spare room’. They were [saying], ‘You’re not going to be homeless, so don’t worry’.

*RuHS-TAHS-4*

In the case of the regional service, with the exception of one group of AHPs, transitional accommodation had not historically been offered to AH staff relocating to take up a position. These staff discussed that it was challenging to find suitable housing and this was made more difficult by house viewings usually being held during work hours.

I just couldn’t find anything. I just thought, ‘I can’t find anything that fits the bill’, and it didn’t matter how many properties people threw under my nose … then it was around Christmas time and Christmas was impossible to find anything. No one will take you on inspections. And trying to find inspections that were on after hours was really difficult. All the real estate agents shut, they open at 8.30, they shut at 5pm. My working hours are anywhere between 7 and 5, so it’s just, it was impossible to even to get to a real estate office to say, ‘I’m looking for a property, I want some support’ … I’d have friends going to inspections for me.

*ReHS-TAHS-10*

New staff interested in shared housing discussed commonly finding housemates through the workplace, either by word of mouth among staff or through the support of their manager.

I had [housing] options in place before I moved … She [her manager] sent an email around, you know, around and then just said, ‘these are all the potentials’. I think there were about six different contact numbers of people to [share with]. That made things so much easier … a lot easier not having to come and try and then find things on my own.

*ReHS-TAHS-1*

### 3.7. Community

#### 3.7.1. Establishing Social Connections, Particularly in the Workplace, is a Priority Issue for Almost all AHPs Relocating for Work

Newcomers without pre-existing social links to the town discussed, in the first instance, relying on their work colleagues for social connection. This tended be team based in the workplace. For the rural health service, this involved the whole community health team. This team had a high proportion of early career newcomers in early adulthood who were mostly single, and these staff members were described as social and inclusive of newcomers:

Pretty much from the first day, for the first week, I felt included like that. Everyone in, particularly in this area, is incredibly welcoming, really amazingly so.

*RuHS-TAHS-6*

In the regional service, social connection tended to be situated in teams/services and/or specific professions. For most teams, the social activities were limited to the workplace and involved shared morning teas or lunches. A couple of teams organised regular out-of-hours social events, but again these teams tended to comprise a large cohort of staff in early adulthood who were mostly single:

[In the] X [profession name] team I felt really welcomed. As soon as I got here, they made sure I was okay, got to know me, had a welcome dinner. Y [staff member’s name] organises all of the social events for X and that was a good opportunity to get to know them outside of work, you talk about different things.

*ReHS-TAHS-9*

In both services, a significant number of AH staff commuted to work and/or spent most weekends in their hometown or travelling to where their partners resided. For these staff, social connection opportunities tended to be limited to activities that were offered in the workplace during work hours.

I would love to be closer and I have close bonds with people [here] but there is still the [distance] barrier that separates you from developing … things further. And a lot of other people are not from here, so they’re most likely to go back home [straight after work] anyway. But everyone’s from different directions and some people have kids and it just gets really messy [trying to catch-up out of work].

*ReHS-TAHS-6*

There was a couple of people there who just weren’t interested in any of the regional stuff, unless it was open after hours on a Monday to Thursday because ‘we’ll only be here for one year and we’ll be going to Melbourne every Friday night and coming back on Monday morning’.

*ReHS-KI-8*

Most new AH staff who relocated for work, especially those who were single and/or in young adulthood, expressed that they were keen to broaden their social connections both in the workplace and in the local community:

I don’t have friends here and I’ve sort of grown distant from my friends from uni. So that’s, like it does impact a lot that I don’t have friends, I don’t have, like, a social life … I do wish there were more opportunities to make friends and more sort of social events, which there’s definitely a lack of in X [town’s name].

*ReHS-TAHS-24*

Even new AH staff returning to live in their hometown expressed interest in making new friends as they found their previous social network had diminished:

Moving back to a small town I thought that I’d know everyone. I don’t know anyone there either… they’ve all moved away.

*ReHS-TAHS-22*

#### 3.7.2. Linking into Local Activities is of Importance for Many AHPs Relocating for Work

Social connection with work colleagues often provided an entrée into the town and the range of activities on offer. This social entrée was described as important for new staff as they commonly found it difficult to find out what events or activities were on or available:

The social activities are quite underground in [town’s name] and so there’s a whole lot of things going on but there’s never any communication about it or even being sort of asked.

*ReHS-KI-2*

Social activities on offer in both towns mostly involved a hospital-based social club, sporting groups, pub trivia nights, music and winery events and a young professionals’ network.

I think my main, I guess, outlook for finding friends has been through work and then through the tennis club and then if you know someone and they bring someone new along then.

*ReHS-TAHS-9*

Such activities were described as being better suited to young adults, extroverts and those from Anglo-Celtic cultural backgrounds.

To be honest, no [not interested in participating in social activities in town], because I’m sort of moving from that social young stage into the settling down, sort of maybe getting married stage. But to be honest, and I’m not a super social person, I’m pretty slack, I’m a bit of homebody as well.

*ReHS-TAHS-8*

They’re fine, they’re nice [other team members], I just, I don’t go out … Yeah see [if] I go out, I don’t drink, I’m Muslim, most of the food they have I can’t have, as [it’s not] halal. It’s not that I don’t make friends here, I’ve got friends here, but I just don’t socialise. Out of hours I don’t socialise that much.

*ReHS-TAHS-35*

In both towns, the opportunities for making new friends in the community were described by many new staff as mostly being sporting groups:

I think in the country towns is if you’re not sort of in the football, netball, then it’s harder I suppose to make those connections outside of work and get to know the people.

*ReHS-TAHS-19*

However, some newcomers who had approached local sporting groups experienced them as unwelcoming and/or cliquey:

I was made aware of a local running group … I eventually made contact with one of the people and he said, ‘Yeah. Come along, get involved.’ I thought it would be good to get involved in that … I’ll give it go…I quite enjoyed it, but … they didn’t tend to come up to me and say: ‘I’m such and such, how you going? What you been doing etc., etc.?’ There wasn’t a lot of that. I haven’t been back.

*RuHS-TAHS-7*

## 4. Discussion

### 4.1. Review of Findings

The emergent themes depict contextual challenges within all of the WoP-RIF domains, most of which negatively impact the attraction, recruitment and retention of AH staff. In both rural public sector health services, AH turnover and workforce shortages were a significant and chronic problem. The AH managers and executive commonly demonstrated a nuanced understanding of the recruitment and retention challenges in terms of the differences between AH disciplines, experience levels, life stage and social factors. However, despite the extent of the problem and a sound understanding the workforce challenges, there were few specific attraction, recruitment and retention strategies in place for the AH workforce. Notable exceptions are the financial and accommodation incentives offered by the rural health service to new health staff needing to relocate in response to the local housing shortage and the identified disincentive of costs associated with relocation. 

This study also identified many examples in both services of poor processes, inefficiencies and inconsistencies in the application of policies and procedures which negatively impacted on the job satisfaction of AH staff. The importance of skilled AH leaders/managers was strongly supported and found to be commonly lacking in the two services. Managers in both services generally had a sound understanding of the significant challenges facing entry-level AH staff and the importance of PD for AH staff. Programs specifically targeting entry-level AH staff and supports for undertaking external PD for all AH staff were in place. However, because of organisational inefficiencies, these were not always accessible to all AH staff. The need for local AH career development opportunities was widely accepted as being essential for medium–long-term retention but very little activity was being undertaken to address this issue. This study highlighted how place-based social processes are an important influencing factor on job retention and this was generally well understood by AH management and executive, but again, very little activity was being undertaken to address the issue and none at all involving the broader community. 

Overall, this study highlighted that public sector rural health services were not adequately addressing AH workforce challenges in an efficient, systematic or strategic manner and there was an urgent need for this occur to stabilise the existing workforce and support the development of a sustainable AH workforce. While this finding was not surprising to either the author or the two partnering health services, what was unexpected was the extent to which the challenges were so similar and that the bulk of the recommendations would be the same for both services.

The findings resonate strongly with other Australian AH rural workforce studies exploring the enablers and barriers to rural recruitment and/or retention [7,14,18]. Of particular interest are the many similarities this study has with the findings in a recent qualitative study investigating AHPs’ transition to practice in rural regions of South Australia involving AHPs (n = 16) and managers/employers in the public sector (n = 2) and the private sector (n = 4) [19]. Kumar et al.’s study categorised transition into ‘before’, ‘during’ and ‘after’ stages. In the ‘before’ stage, comparable findings relate to ‘job availability’, with AHPs discussing the need to ‘get experience’ and the difficulties in getting a job as a new graduate, and managers/employers discussing lengthy recruiting processes outside their locus of control (related to higher organisational departments) negatively impacting recruitment [19]. In the ‘during’ stage, an analogous finding was the nature of rural practice (e.g., staffing shortages, small AH teams, lack of experienced staff) and the related challenges of providing mentoring/clinical supervision and accessing PD given the workplace environment. The challenges of rural practice environment were identified by both AHPs and managers/employers as contributing to almost all the AHPs feeling a lack of support in transitioning to the job. This was also found in the present study and has generally been well identified in the extant rural health workforce research [10,20,21,22]. In Kumar et al.’s study, working in a supportive team was found to be an important aspect of supporting transition and deciding to stay and again this was well supported in this study and other AHP rural retention literature [19,23,24,25]. The Kumar et al. study, as well as others, identified that incentives such as accommodation support may help attract AHPs to ‘go rural’ but these are not as important as access to PD and do not influence retention [18,19,26]. In the Kumar et al. study, ‘social/lifestyle’ was a critical factor identified by employers/managers for successful transition and retention of AHPs. This involved different factors in the stages of transition including ‘before’ (recruiting)—the need to assess AHPs personality types and the likelihood of ‘fitting in’; ‘during’—the significance that social networks in the workplace play in social inclusion; and ‘after’(retention)—the need for AHPs to be embedded within the community with established connections with local people and groups [19]. 

The critical role social/lifestyle factors play in successful transition and in supporting retention of AHPs in rural positions is increasingly being recognised in the extant literature (including by this author) and understanding is rapidly developing as to what processes are at play and which are modifiable [7,26,27,28,29,30,31]. Kumar’s findings relating to social/lifestyle dimensions to retention are equivalent to the WoP-RIF community/place domain. This domain was recently explored in Cuesta-Briand et al.’s Western Australian study of factors influencing junior doctors’ (n = 21) career decision making [32]. In their study, two key themes were identified: the importance of place and people, and broader context factors. Place and people factors resonate strongly with the present study’s findings involving the community/place domain. In regard to ‘place’, junior doctors with a strong rural intention discussed lifestyle factors associated with a particular place, and the importance of this place providing a sense of community. Respondents in this study also considered place to include the workplace and the need for colleagues to be friendly and supportive [32]. In regard to ‘people’, the physical settings (both town and workplace) were identified as being intrinsically linked to the people inhabiting them and connectedness was important [32]. Accommodating life partners’ careers was perceived as a main barrier to attracting and retaining doctors in rural places [32]. This was upheld in the present study. While the place and people processes were congruent with this study’s findings, the broader context factors were dissimilar. Concerning the junior doctors’ thoughts regarding career opportunities, a commonly held viewpoint was that they were limited to primary care and general practice in rural places and that other medical specialisations would require them to train in an urban setting [32].

Humphreys, Wakerman and Wells argue that a sustainable rural health system requires a sustainable ‘fit-for-purpose’ health workforce [33]. To achieve this, policies that support an integrated training pipeline for all the health professions as well as an ‘effective, flexible, bundled retention strategy’ [34] are needed. The author argues that the latter is always contextual and a redistribution of Australia’s health funding is needed at both national and state levels to allow health services and communities to implement strategies that can respond to the particular local challenges and opportunities affecting the recruitment and retention of health staff. Rigorous evaluation of these local endeavours may assist in identifying successful initiatives that have potential to be scaled up and contribute to the evidence-base for other health services and communities to use, as well as generally strengthen Australia’s rural health system [34]. The next part of this research study is an evaluation involving analyses of the recommendations’ utility for improving AH retention by two Victorian rural public health services. The outcomes and conclusions drawn from this stage of the research are forthcoming.

### 4.2. Analysis of Recommendations

To analyse the 10 recommendations (listed in Table 4), this study draws on two key studies presenting evidence-based recommendations to improve attraction, recruitment and retention of rural and remote workers: the World Health Organisation (WHO) [35] and Buykx et al. (2010) [36]. The analysis also draws on other rural health workforce literature where relevant.

#### 4.2.1. Organisational/Workplace Domain

A key challenge identified as impacting the attraction of AHPs related to housing concerns and financial costs of relocating. In line with various WHO and Buykx et al. recommendations, the author recommended that transitional accommodation and reimbursement of relocation costs be routinely offered to AHP candidates needing to relocate for work (Recommendation 1). 

Other Australian rural workforce studies have argued that ‘work systems’ need to suit the particular work environment and that local managers need to be able to develop employment policies that are responsive to the local context [3,4]. To improve the attraction for AHPs who are the ‘right person’ for the work and place context, this study identified the need to strengthen existing recruitment materials by better promoting the work benefits and local lifestyle and living features, which is in line with Buykx et al.’s recommendation to maintain adequate and stable staffing (Recommendation 2). 

The WHO identified that workplaces needed to meet an ‘acceptable standard’ and Buykx et al. recognised the importance of health services being perceived as ‘efficient’ organisations and that health workers’ initial entrée to the service can influence their perception about the suitability of the job and retention. Thus, streamlining the HR processes was recommended (Recommendation 3). 

In a study of Australian remote health services, line managers were seen by health staff as representing the ‘organisation’ and their level of support was equated with what the organisation provides [22]. Therefore, the need to support strategic and effective AH leadership was recommended (Recommendation 4).

#### 4.2.2. Role/Career Domain

Entry-level AHPs were found to experience a difficult transition to work and those in early adulthood (early–mid 20s) who had relocated for work were found to be the most vulnerable to experiencing social disconnection and loneliness [17]. Therefore, a support program to assist entry-level AHPs to adjust to work, build their clinical confidence, support their professional and career development, and foster social connection was recommended (Recommendation 5). 

The importance of health workers’ professional identity for their job satisfaction and thus retention is widely recognised and both the WHO and Buykx et al. recommend professional development. For those AHPs working in rural and remote health services, given their more limited staff numbers, having regular access to profession-specific PD is particularly important for reducing professional isolation. Therefore, the author recommended reviewing the service’s AH PD policy to ensure equity of access for staff (Recommendation 6). 

Both the WHO and Buykx et al. identified the importance of career advancement for retention. Development of an AH career pathways program was recommended (Recommendation 7).

#### 4.2.3. Place/Community Domain

The importance of place and community were identified and addressed in recommendations 8 and 9. Buykx et al. identified the need for social and community support for new staff and their family members, while the WHO identified that living conditions had a significant influence on both rural attraction and retention and this included housing, employment opportunities for partners, adequate schools, road access and internet connectivity. Other AH rural workforce studies have identified the need for rural health staff to have meaningful social connections in place for medium–long-term retention [18,37]. In WoP-RIF, these social and community factors were included under the community and place domain. Key elements included 1) having strategies in the workplace and in-community to welcome and support the initial adjustment of new staff and any family members, 2) local town residents being welcoming and accepting of newcomers, and 3) the active involvement of local community organisations to run activities/events that support the social integration of newcomers [14].

### 4.3. Broader Relevance of the Recommendations 

This study’s recommendations relating to the community/place domain will likely have generalisability for the broad health workforce in other high-income countries, especially those that have similar Westernised health, education, social and training systems, such as Canada, United Kingdom and United States. This is supported by research conducted in high-income countries across different rural contexts and health professions where matters relating to people and place (including supportive work environments) are often identified as being of high importance in attracting and retaining health professionals [30,32,38,39,40]. Further exploration is needed as to whether these community/place recommendations could have relevance for rural-based health professionals from low-income countries given the differences in cultures and health and education systems [41,42]. On the other hand, the recommendations made relating to organisational/workplace and role/career domains are likely highly contextual and relate specifically to Australia’s AHPs working in public sector services. In this circumstance, salaries and work conditions are collectively set under an EBA and did not feature as impacting either recruitment or retention. In the case of rural medical professionals (i.e., general practitioners) in high-income countries, most work in private practice and their earning potential is variable. For this group, income and work conditions are major factors for attraction and retention [38,39]. In addition, recruitment of rural doctors may be influenced by financial enticements such as bonded placements, loan repayment schemes or other financial incentives, and these types of financial benefits are less commonly on offer to AHPs and nurses [41,42].

The author supports the WHO’s position that a sustainable rural health workforce requires incentives and interventions that are attractive to individual health professionals [43]. This requires that health professionals’ ‘reality’ is well understood, including the education and health systems and workplaces in which they are trained and/or work [43,44]. Thus, in the case of organisation/workplace and role/career domains, these ‘realities’ will likely markedly differ between health professional groups (allied health, medicine, nursing). In addition to needing to address the differing realities between the health professions, effective incentives and interventions must also be able to flexibly respond to the fact that the three domains are interlinked and career aspirations and quality of life needs will change over the life course. 

### 4.4. Limitations

This study was conducted in one geographical location (Victoria) in Australia’s least geographically remote state and limited to two public health services, which may limit the transferability of the findings. While this study provides rich data on the issues and concerns experienced in the first 12 months of working in a rural position, the AHP interviews were undertaken at just one point in time, while it is known that influences on retention change over time. To better understand retention and the impact of individual factors, longitudinal studies of rural-based AHPs applying quantitative measures and in-depth qualitative research at particular time points are needed.

## 5. Conclusions

The findings from this study highlight that there are many shared organisational and workplace challenges that contribute to poor recruitment and ‘avoidable’ AH staff turnover. To support a sustainable AH workforce, rural public sector health services must be efficient and demonstrate strategic leadership and vision. In this context, efficiency means such things as improving recruitment processes and ensuring that PD programs are accessible to all staff, while strategic leadership and vision mean going beyond just understanding AH workforce challenges and taking action to develop local programs, opportunities and supports that allow AH staff to thrive and grow in place. This includes understanding the critical importance of PD and career advancement and working to address challenges and create local opportunities for AH staff at all grade levels. It also requires a systematic approach to addressing the social needs of AH workers who have relocated for work and addressing the differing social support needs individuals have depending on their life stage, relationship status and culture. Strategic leadership and vision include taking a whole-of-community approach to effectively support individual health workers and their family members to successfully adjust to a new place and develop a sense of belonging in place. 

Given the strength of the findings that underpin the 10 shared recommendations developed for the two rural health services, the author contends that these approaches will likely have utility for other rural public sector health services in high-income countries. These recommendations provide guidance for the development of recruitment and retention strategies aimed at achieving a more stable and sustainable AH workforce. Furthermore, the recommendations relating to the community/place domain will likely benefit the broader rural health workforce in other high-income countries.

## Figures and Tables

**Table 1:** Influences on health staff’s job/personal satisfaction in the Whole-of-Person Retention Improvement Framework.

WoP-RIF Domains	Major Influence on Job/Personal Satisfaction
Workplace	High-quality workplace relationships with line manager and in team
Organisational	Organisation managed efficiently and strategically
Role	Opportunities to engage with other discipline-specific health professionals and governing bodies
Career	Opportunities for career development/advancement
Place	Experience a sense of belonging in place
Community	Community involved in the planning and implementation of recruitment and retention strategies

**Table 2:** Overview of participants by AH profession or position type.

AH Profession/Position Type	Regional Health Service	Rural Health Service	Total
**No. Target AH Staff Participants**			
Allied health assistance	4	-	4
Dentistry	NA	2	2
Dietetics	2	2	4
Exercise physiology	2	1	3
Medical laboratory science	2	-	2
Diagnostic imaging medical physics	9	1	10
Occupational therapy	3	3	6
Podiatry		2	2
Pharmacy	2	-	2
Physiotherapy	9	2	11
Psychology	1	-	1
Social work	1	1	2
Speech pathology	2	-	2
	37	14	51
**Key Informant Participants**			
Executive	1	3	4
AH managers	10	3	13
Other health managers	1	1	2
AH locums	4	-	4
	16	7	23
All participants	53	21	74

**Table 3:** The key identified themes and elements perceived and experienced as impacting the recruitment and retention of allied health professionals (AHPs).

WoP-RiF Domains	Key Identified Themes	Key Elements
Workplace	Degree of challenge attracting, recruiting, and retaining AHPs varies depending on profession, experience level and life stage	Differing levels of recruiting challenge between AH disciplines. AHPs from over-supplied professions commonly experience more job insecurity. For entry-level AHPs, retention NOT recruitment is the main challenge as a result of limited professional development and career building prospects and wanting new experiences and more social opportunities. For experienced AHPs, recruitment NOT retention is the primary challenge and is attributable to life stage and social disincentives. Offering financial incentives for relocation to reduce/eliminate out-of-pocket expenses and accommodation uncertainty is important for attracting candidates and for successful recruitment. In rural services, turnover of AH staff is fairly constant and has a high level of direct and indirect costs, particularly regarding the workloads of team members.
	AH managers usually recruited from existing workforce and poorly prepared for leadership	In rural services AH managers are commonly promoted from the existing pool of AH staff. The leadership skill level of AH managers, especially those recently promoted, is often very poor. This was explained by university training being focused on developing clinical skills and managers receiving limited on the job support or training.
Organisational	Overly complex human resources systems negatively impact successful AH recruitment and burdensome for AH managers	Human resources recruitment systems are very compliance focused and commonly result in onboarding delays of new staff. Slow onboarding has been experienced by managers (e.g., losing the preferred candidate). High AH turnover, combined with poor human resources processes, negatively impacts managers in terms of additional workload and stress.
Role	Most entry-level AH staff experience a challenging adjustment	Entry-level AHPs have a very challenging initial adjustment to their first AH job in terms of managing the size and demands of the job and their confidence as clinicians. Experiencing one’s line manager and other team members as being supportive is critical for the successful adjustment of entry-level AHPs. The types of adjustment challenges, benefits and opportunities facing entry-level AHPs differ depending on the size of the health service’s AH workforce.
	Professional development opportunities are a high priority for AHPs and the level and type of support offered is not always well understood by AH staff or consistently implemented by AH managers	Access to, and organisational support for undertaking, professional development is important for all AHPs but especially those in early career. Among AH staff, it was often unclear what external PD was being/could be supported by the service and it was frequently differently applied by AH line managers. The level of organisational support for PD and financial support offered to AH staff significantly differed between the two services, with rural service offering support well above the regional service.
Career	Limited career development/advancement opportunities for AHPs working in rural services	AH staff observed that those in senior clinical roles and/or managers tended to stay in their roles for the medium to long term and so there were few opportunities for clinical career advancement in the service. The lack of AH career advancement opportunities was considered a major obstacle for improving AH staff retention.
Place	Securing suitable housing is a priority issue for all AHPs relocating for work	AHPs relocating for work to the health services commonly experienced housing challenges in the respective towns. The level of organisational support for transitional accommodation and securing private housing offered to AH staff significantly differed between the two services, with the rural service offering support well above the regional service. The workplace and suggestions/support offered by other staff and managers were the most important source of information for potential housing and housemates.
Community	Establishing social connections, particularly in the workplace, is a priority issue for nearly all AHPs relocating for work	Work colleagues are often relied on for initial social connection. Social support in the workplace tends to be team based and occurs differently depending on the size of the health service and the number of teams and the physical location of the teams. Many AH staff commute to work and/or spend most weekends away, and so their social connection opportunities are limited to the workplace and work hours. Almost all staff relocating for work, even those who were returning home, were keen to expand their social connections but those in early adulthood and/or unpartnered were by far the most enthusiastic.
	Linking into local activities is of importance for many AHPs relocating for work	Reliance on work colleagues for initial social connection often provided an entrée into the town and the range of activities on offer, which otherwise were hard to find. The types of social activities on offer in both towns were considered to be fairly limited (e.g., sporting pubs/entertainment venues) and tended to suit young adults, extroverts and those from Anglo-Celtic cultural backgrounds. Sporting clubs offered the most opportunities for social connection but were not always welcoming of newcomers.

**Table 4:** Ten common recommendations to improve retention of AH workforce in two rural public sector health services and their correspondence to recommendations made by the WHO and/or Buykx et al.

WoP-RiF Domains	Key Themes Identified	Study Recommendations	WHO Recommendations [35]	Buykx et al. Recommendations [36]
Organisational	Degree of challenge attracting, recruiting, and retaining AHPs varies depending on profession, experience level and life stage	Ensure new staff needing to relocate for work are routinely offered paid transitional accommodation and reimbursement of relocation costs. Identify the main attractors/detractors impacting the successful recruitment and retention of AH staff and develop marketing materials that promote the benefits and opportunities for use in recruiting.	Make it worthwhile to move to a remote or rural area Pay attention to living conditions	Maintaining realistic and competitive remuneration—packaging benefits Providing appropriate and adequate infrastructure—adequate housing Maintaining adequate and stable staffing
	Overly complex human resources systems negatively impact successful AH recruitment and burdensome for AH managers	3. Streamline HR systems and recruiting processes to support faster recruitment/onboarding of new AH staff.	Ensure the workplace is up to an acceptable standard	Fostering an effective and sustainable workplace organisation
Workplace	AH managers usually recruited from existing workforce and poorly prepared for leadership	4. Ensure AH managers have an evidence-based understanding of the factors influencing recruitment and retention of AHPs in rural health services and, in particular, the importance of their being skillful leaders and supportive managers.		Maintaining adequate and stable staffing
Role	Most entry-level AH staff experience a challenging adjustment	5. Establish a two-year early career AH support program to assist entry-level staff manage the size and demands of the job, develop their clinical skills, provide support for professional development and career development and support social connection in the workplace.	Facilitate professional development	Shaping the professional environment that recognises and rewards individuals making a significant contribution to patient care
	Professional development (PD) opportunities are a high priority for AHPs and the level and type of support offered is not always well understood by AH staff or consistently implemented by AH managers	6. Review the AH PD policy and develop a system that is consistent and equitable for all AH staff.		
Career	Limited career development/advancement opportunities for AHPs working in rural services	7. Build AH managers’ understanding of the range of AH career pathways (both clinical and non-clinical) to assist them in better supporting the career development of their staff.	Design career ladders for rural health workers	
Place	Securing suitable housing is a priority issue for all AHPs relocating for work	8. Work with community organisations to establish a strategy for professionals relocating to the town/region to feel welcomed and to assist with addressing initial adjustment needs (e.g., housing, local doctor, vet, hairdresser, dentist).	Pay attention to living conditions	Ensuring social, family and community support
Community	Establishing social connections, particularly in the workplace, is a priority issue for almost all AHPs relocating for work	9. Ensure there are AH workforce-wide policies and systems in place in to welcome and support the social connection of new AH staff.	NA	
	Linking into local activities is of importance for many AHPs relocating for work	10. Work with community organisations to establish a strategy for professionals relocating to the town/region to welcome and encourage social connection and belonging in place.	NA	
